# Supplementary material for: Rational Identification of Ritonavir as IL-20 Receptor A Ligand Endowed with Antiproliferative Properties in Breast Cancer Cells
Source: Int J Mol Sci. 2025 Feb 2;26(3):1285. doi: 10.3390/ijms26031285 (PMC11818535; doi:10.3390/ijms26031285)
Supplement: Supplementary file 1 [file ijms-26-01285-s001.zip › ijms-3408325 Supplementary_materials.pdf]

(a)

	Docking scores (kcal/mol)					
	BS-A	BS-D	MW	logP	Molecular target	Therapeutic application
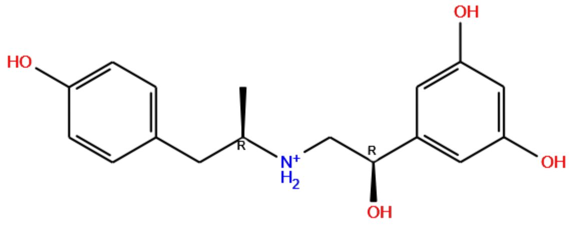<br>Fenoterol	-7.59	-6.83	303.35	1.36	β_2 adrenergic receptor	Asthma

(b)

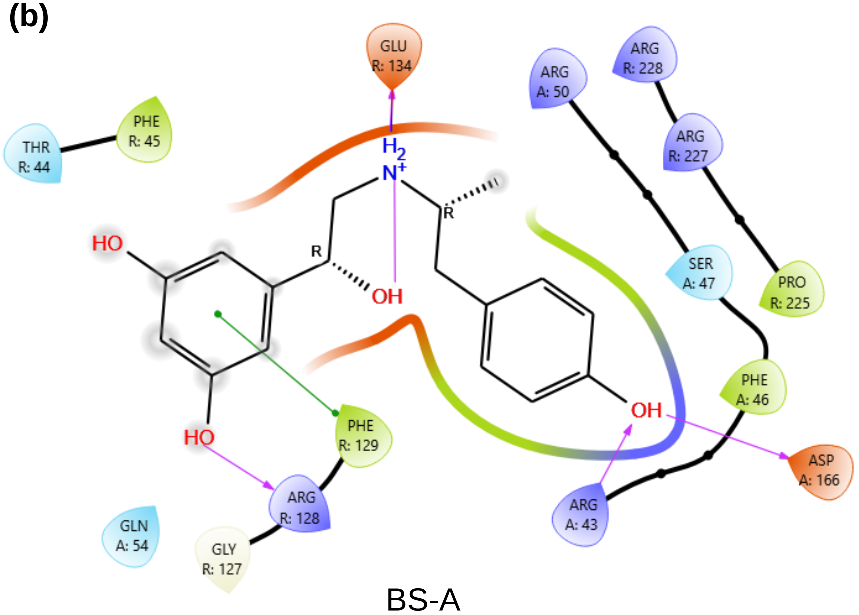

BS-A

(c)

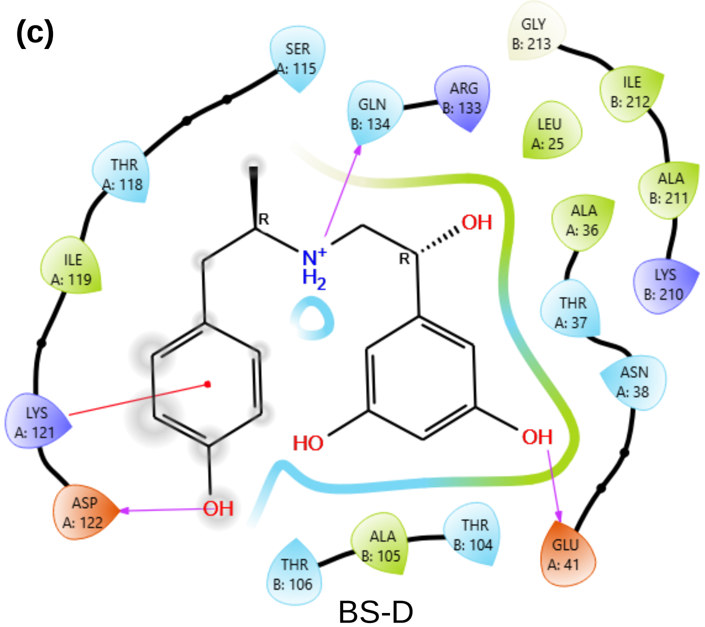

BS-D

Figure S1. (a): 2D structure and docking score (kcal/mol), Molecular Weight (Da), log P, Molecular Target and Therapeutic application of the identified ligand [36,44]. (b) and (c): 2D ligand interactions of Fenoterol within binding sites BS-A and BS-D, respectively.

(a)

	Docking scores (kcal/mol)					
	BS-B	BS-F	MW	logP	Molecular target	Therapeutic application
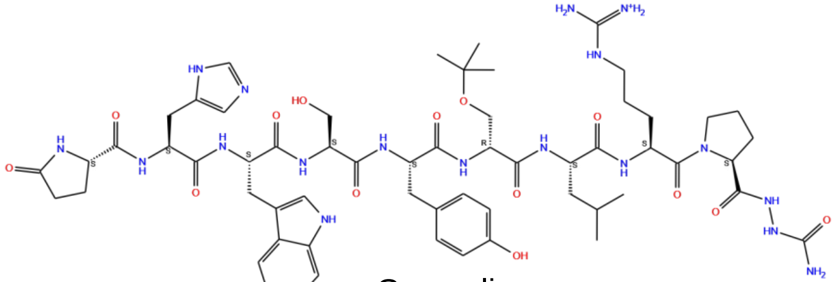 Goserelin	-8.151	-8.054	1269.41	0.3	GnRH	Hormone-dependent cancers

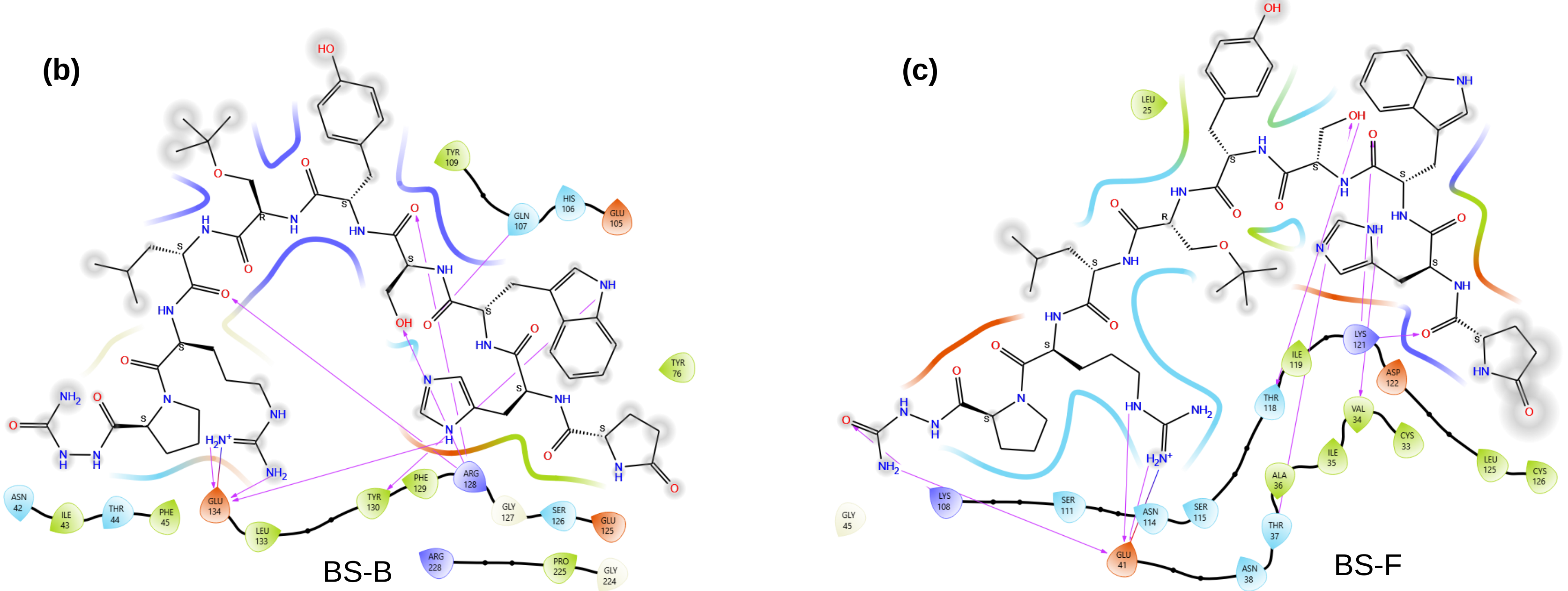

Figure S2. 2D structure and docking score (kcal/mol), Molecular Weight (Da), log P, Molecular Target and Therapeutic application of the identified ligand (a) [36,40,41]. 2D ligand interactions of Goserelin within binding sites BS-B (b) and BS-F(c). GnRH: Gonadotropin Releasing Hormone.

(a)

	Docking scores (kcal/mol)					
	BS-C	BS-E	MW	logP	Molecular target	Therapeutic application
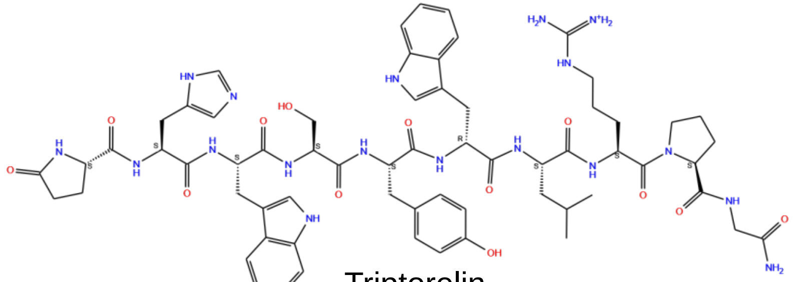 Triptorelin	-8.02	-7.38	1311.47	1.07	GnRH	Hormone-dependent cancers

(b)

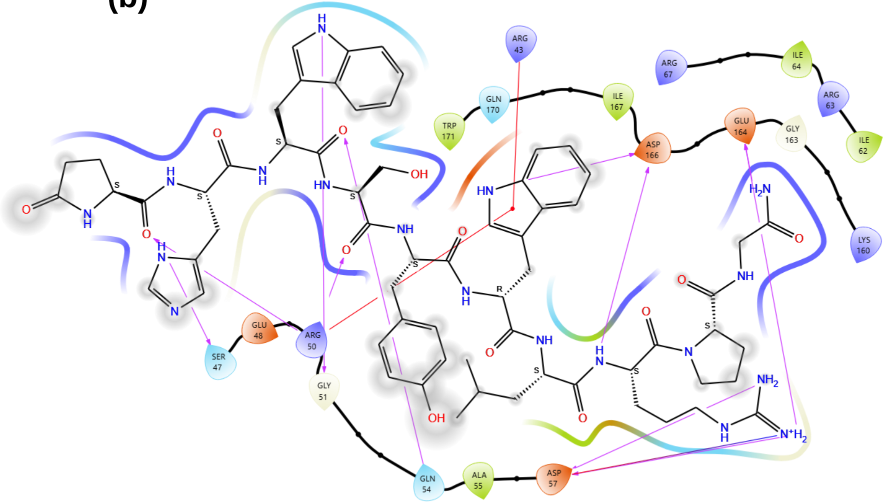

BS-C

(c)

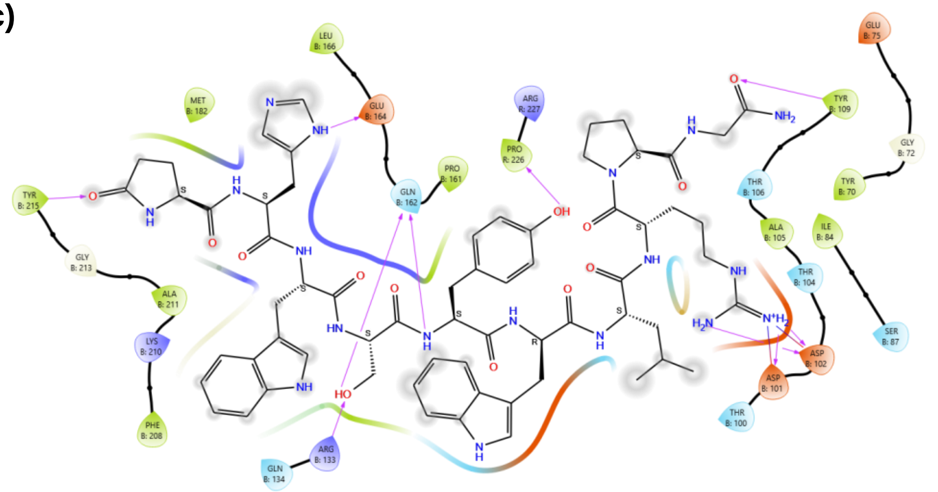

BS-E

Figure S3. 2D structure and docking score (kcal/mol), Molecular Weight (Da), log P, Molecular Target and Therapeutic application of the identified ligand (a) [36,42,43]. 2D ligand interactions of Triptorelin within binding sites BS-C (b) and BS-E (c). GnRH: Gonadotropin Releasing Hormone.

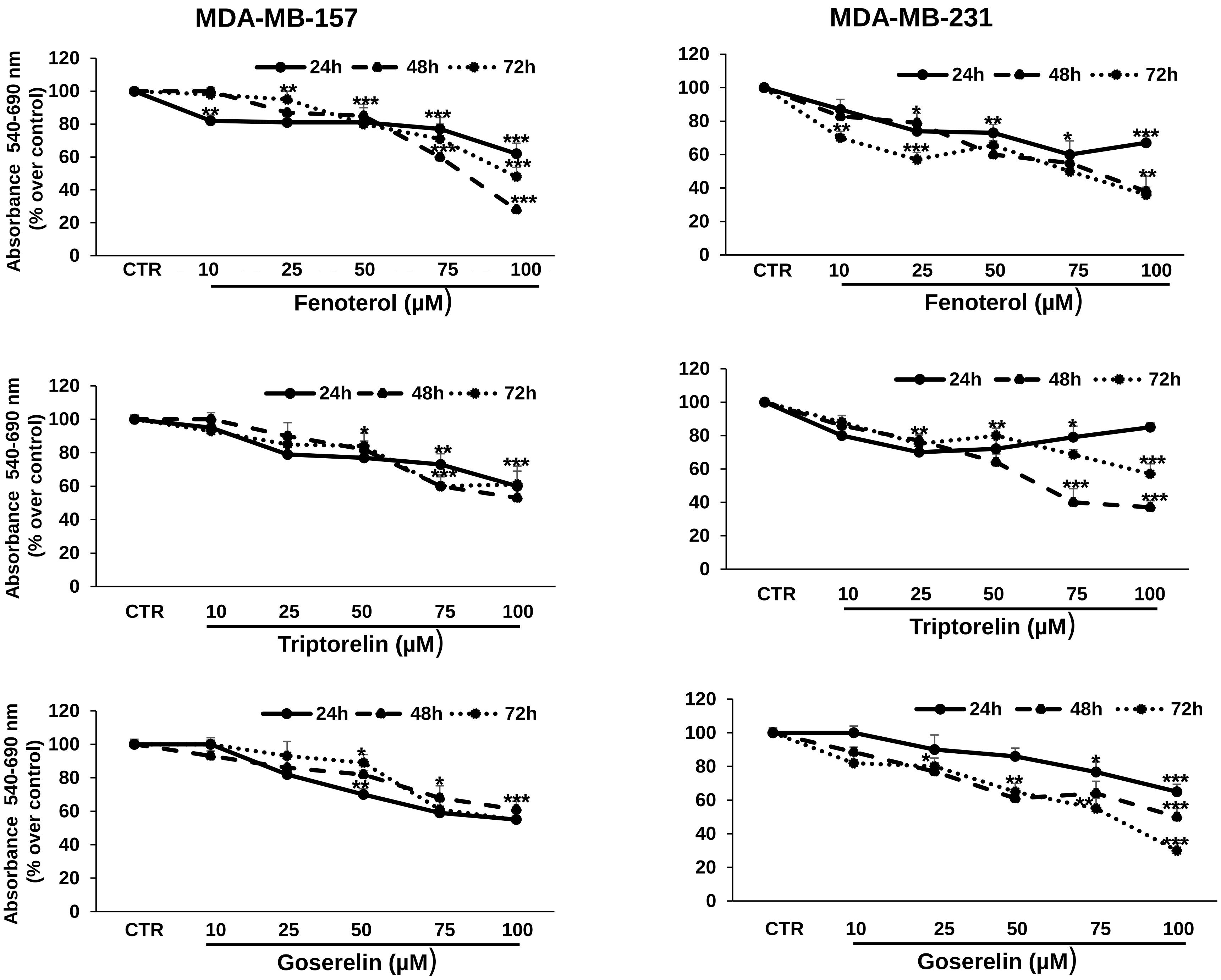

Figure S4. Effects of Fenoterol, Triptorelin and Goserelin on cell viability of MDA-MB-157and MDA-MB-231 cells. Cell viability evaluated by MTT after 24, 48 and 72 h of incubation with different concentrations of Fenoterol, Triptorelin and Goserelin. Results are presented as mean ± SD of three independent experiments performed in eigthplicate. Statistical analysis was performed using the Tukey-Kramer multiple comparisons test. *$p<0.05$, **$p<0.01$, ***$p<0.001$ *vs* control. Control is indicated as CTR.

Table S1. 2D structures and docking scores (kcal/mol) of the identified ligands within binding sites BS-A, BS-B, BS-C and BS-D.

	Docking scores (kcal/mol)			
	BS-A	BS-B	BS-C	BS-D
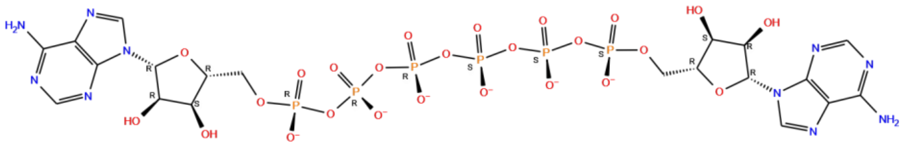 ZINC000256824195	-9.46	-7.68	-7.55	-8.86
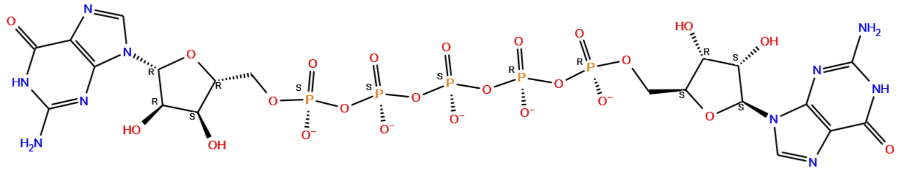 ZINC000261498054	-10.28	-7.68	-7.56	-8.55
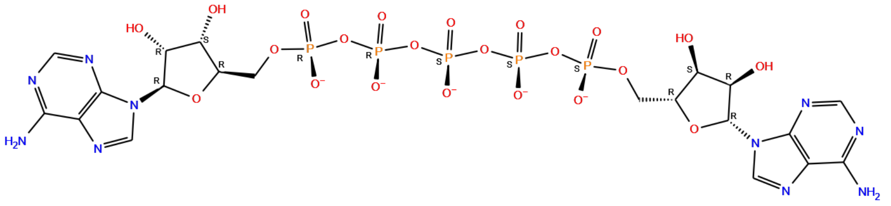 ZINC000096085195	-10.05	-7.95	-6.59	-8.68

Table S2. 2D structures and docking scores (kcal/mol) of the identified ligands within binding sites BS-C, BS-F, BS-B and BS-E.

	Docking scores (kcal/mol)			
	BS-C	BS-F	BS-B	BS-E
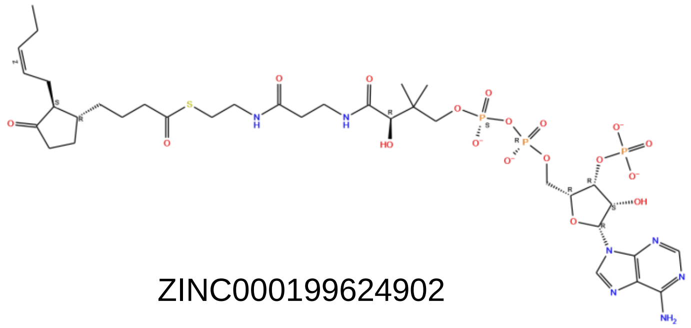 ZINC000199624902	-6.52	-7.89	-7.72	-7.52
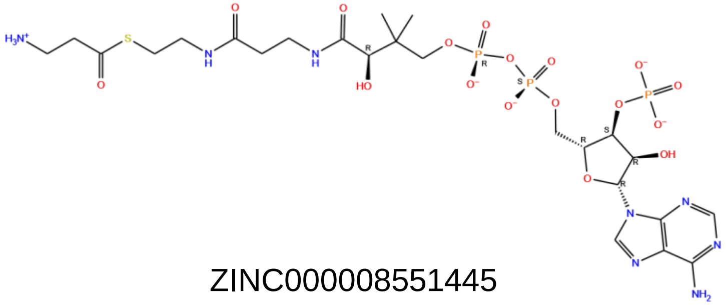 ZINC000008551445	-6.13	-7.64	-6.89	-7.86

Table S3. 2D structure and docking score (kcal/mol) of the identified ligand within binding sites BS-A, BS-D, BS-C, BS-F and BS-B.

	Docking scores (kcal/mol)				
	BS-A	BS-D	BS-C	BS-F	BS-B
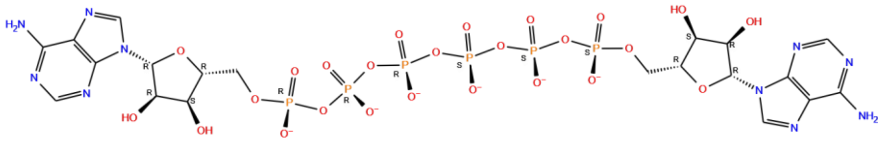 ZINC000256824195	-9.46	-8.86	-7.55	-7.64	-7.68

Table S4. 2D structure and docking score (kcal/mol) of the identified ligand within binding sites BS-A, BS-B, BS-C and BS-E.

	Docking scores (kcal/mol)			
	BS-A	BS-B	BS-C	BS-E
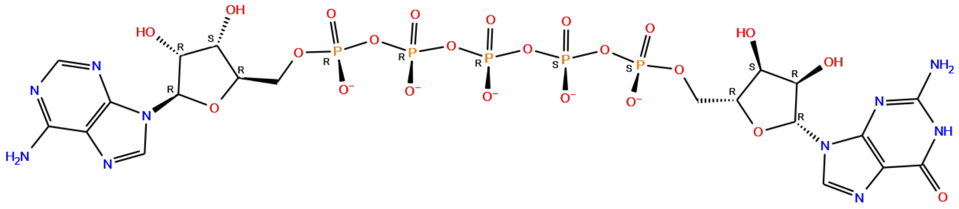 ZINC000261495500	-9.67	-8.02	-7.02	-7.69

Table S5. 2D structure and docking score (kcal/mol) of the identified ligand within binding sites BS-A, BS-B, BS-C and BS-F.

	Docking scores (kcal/mol)			
	BS-A	BS-B	BS-C	BS-F
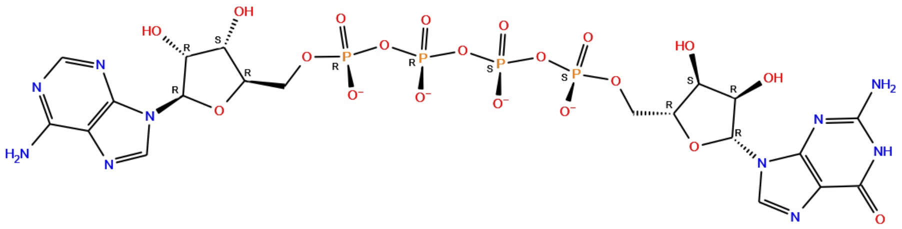 ZINC000096014977	-9.34	-7.28	-6.78	-8.29

Table S6. 2D structure and docking score (kcal/mol) of the identified ligand within binding sites BS-A, BS-B and BS-C.

	Docking scores (kcal/mol)		
	BS-A	BS-B	BS-C
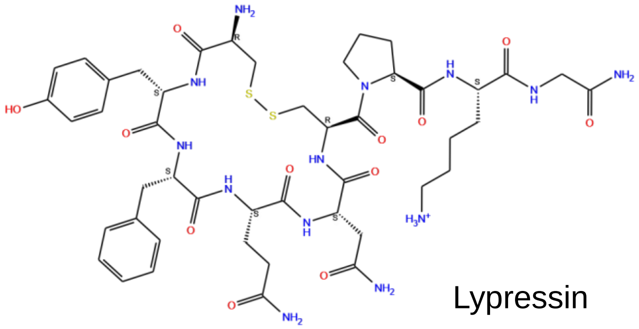 Lypressin	-9.56	-8.34	-7.28

Table S7. 2D structure and docking score (kcal/mol) of the identified ligand within binding sites BS-C, BS-F, BS-B and BS-E.

	Docking scores (kcal/mol)			
	BS-C	BS-F	BS-B	BS-E
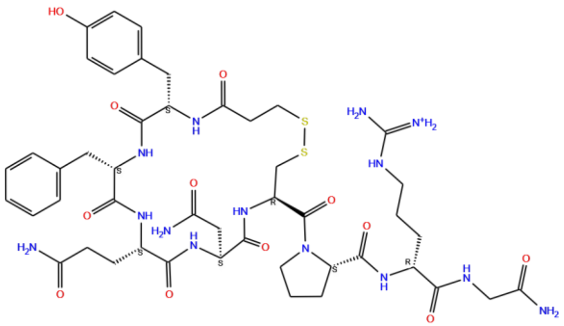 Desmopressin	-7.39	-7.47	-7.59	-8.84

Table S8. 2D structure and docking score (kcal/mol) of the identified ligand within binding sites BS-C and BS-E.

	Docking scores (kcal/mol)	
	BS-C	BS-E
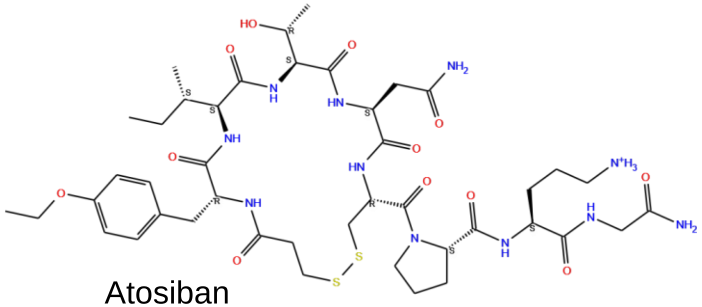<br>Atosiban	-7.55	-8.04

Table S9: Raw data of cell viability assays reported in Figure 5 (a). Each value represents the eightplicates' average of three independent experiments (indicated as Exp.)

MDA-MB-157	CTR	RITONAVIR 10 µM	RITONAVIR 25 µM	RITONAVIR 50 µM	RITONAVIR 75 µM	RITONAVIR 100 µM
Exp.1 24h	0,146	0,130	0,121	0,090	0,073	0,042
Exp.2 24h	0,142	0,120	0,113	0,082	0,064	0,049
Exp.3 24h	0,141	0,123	0,118	0,084	0,060	0,050
Exp.1 48h	0,250	0,210	0,200	0,121	0,045	0,028
Exp.2 48h	0,260	0,209	0,206	0,115	0,052	0,021
Exp.3 48h	0,242	0,205	0,200	0,110	0.041	0,018
Exp.1 72h	0,399	0,321	0,340	0,052	0,021	0,010
Exp.2 72h	0,410	0,316	0,338	0,065	0,019	0,011
Exp.3 72h	0,417	0,319	0,333	0,069	0,022	0,080

MDA-MB-231	CTR	RITONAVIR 10 µM	RITONAVIR 25 µM	RITONAVIR 50 µM	RITONAVIR 75 µM	RITONAVIR 100 µM
Exp.1 24h	0,130	0,124	0,118	0,079	0,058	0,040
Exp.2 24h	0,127	0,116	0,101	0,084	0,065	0,044
Exp.3 24h	0,135	0,118	0,109	0,088	0,060	0,038
Exp.1 48h	0,224	0,194	0,198	0,110	0,038	0,030
Exp.2 48h	0,220	0,184	0,188	0,097	0,036	0,029
Exp.3 48h	0,248	0,200	0,210	0,114	0,045	0,036
Exp.1 72h	0,396	0,320	0,320	0,093	0,054	0,012
Exp.2 72h	0,388	0,318	0,325	0,097	0,063	0,017
Exp.3 72h	0,400	0,312	0,320	0,093	0,058	0,015

Rational identification of Ritonavir as IL-20 receptor A ligand endowed with antiproliferative properties in breast cancer cells

Valentina Maggisano[1†], Adriana Gargano[1,2†], Jessica Maiuolo[1], Francesco Ortuso[1,3], Francesca De Amicis[4], Stefano Alcaro[1,2,3*], Stefania Bulotta[1]

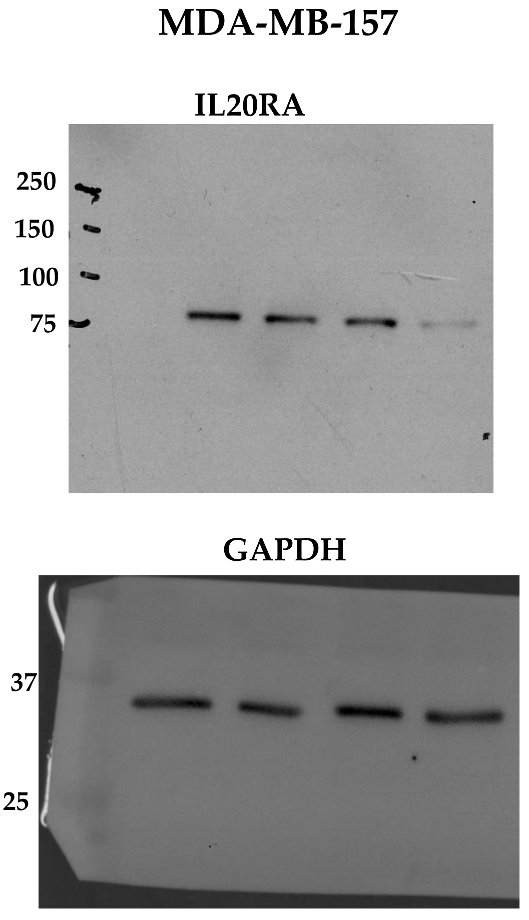

Figure 7a

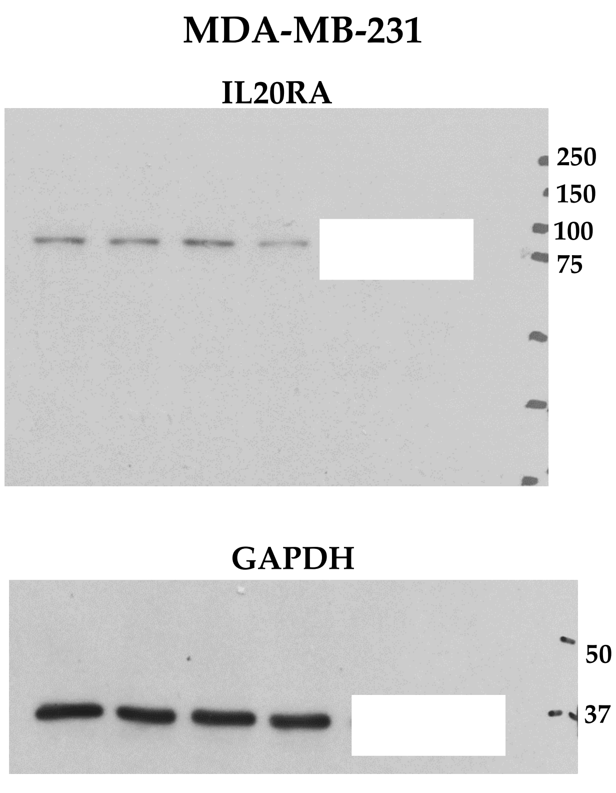

Figure 7a

Original uncropped western blotting of images reported in: (a), Figure 7 (the white shadow covered 2 samples out of this study).
